# A New GlyT2 Variant Associated with Hyperekplexia

**DOI:** 10.3390/ijms26146753

**Published:** 2025-07-14

**Authors:** Jorge Sarmiento-Jiménez, Raquel Felipe, Enrique Núñez, Alejandro Ferrando-Muñoz, Cristina Benito-Muñoz, Federico Gago, Jesús Vázquez, Emilio Camafeita, Emma Clement, Brian Wilson, Beatriz López-Corcuera

**Affiliations:** 1 Departamento de Biología Molecular, Instituto de Biología Molecular (IUBM), Centro de Biología Molecular “Severo Ochoa”, Consejo Superior de Investigaciones Científicas-Universidad Autónoma de Madrid, 28049 Madrid, Spain; jsarmiento@cbm.csic.es (J.S.-J.); raquel.fm1994@gmail.com (R.F.); enrinubal@gmail.com (E.N.); aferrando@salud.madrid.org (A.F.-M.); cristina.benito.munoz@gmail.com (C.B.-M.); 2 Department of Biomedical Sciences, University of Alcalá (UAH), Alcalá de Henares, 28805 Madrid, Spain; federico.gago@uah.es; 3 Centro Nacional de Investigaciones Cardiovasculares (ISCIII), 28029 Madrid, Spain; jesus.vazquez@cnic.es (J.V.); ecamafeita@cnic.es (E.C.); 4 CIBER de Enfermedades Cardiovasculares (CIBERCV), 28029 Madrid, Spain; 5 North East Thames Regional Genetics Service, Great Ormond Street Hospital for Children NHS Foundation Trust, London WC1N 3JH, UK; emmaclement@nhs.net; 6 Northern Genetics Service, Newcastle upon Tyne Hospitals NHS Trust, International Centre for Life, Central Parkway, Newcastle upon Tyne NE1 3BZ, UK; brian.wilson9@nhs.net; 7 Biosciences Institute, International Centre for Life, Newcastle University, Newcastle upon Tyne NE1 3BZ, UK; 8 IdiPAZ-Hospital Universitario La Paz, 28046 Madrid, Spain

**Keywords:** hyperekplexia, GlyT2 variant, glycine transport, lipid raft, proteome, UPR

## Abstract

Hyperekplexia (OMIM 149400), a sensorimotor syndrome of perinatal clinical relevance, causes newborns to display an energic startle reflex in response to certain trivial stimuli. This condition can be lethal due to apnea episodes. The disease is caused by a blockade of glycinergic neurotransmission. Glycinergic interneurons preserve their identity by the activity of the surface glycine transporter GlyT2, which supplies glycine to presynaptic terminals to maintain glycine content in synaptic vesicles. Loss-of-function mutations in the GlyT2 gene (*SLC6A5*) cause a presynaptic form of human hyperekplexia. Here, we describe a new GlyT2 variant found in an infantile patient diagnosed with hyperekplexia. A missense mutation in the open reading frame of the GlyT2 gene inherited in homozygosity caused the substitution G449E in a residue highly conserved across the phylogenetic scale. The sequences of the glycine receptor genes *GLRA1* and *GLRB* did not show abnormalities. We expressed the recombinant GlyT2 variant in heterologous cells and analyzed its pathogenic mechanism. The transporter was totally inactive, behaving as a bona fide loss-of-function mutant. Furthermore, the mutation promoted the abnormal insertion of the protein into the membrane, leading to its large incorporation into lipid rafts. However, there was no apparent alteration of wild-type trafficking upon mutant coexpression, as the mutant was prematurely degraded from the endoplasmic reticulum. Rescue with chemical chaperones was not possible for this mutant. Proteomics demonstrated that the expression of the mutant induced the unfolded protein response and interfered with raft-dependent processes. Therefore, the new variant causes a loss of function regarding GlyT2 activity but a gain of function as a cell proteostasis disturber.

## 1. Introduction

Hyperekplexia, or “startle disease” (OMIM 149400), is a rare neurological syndrome characterized by a pathological alteration of the brainstem startle reflex regulated by glycinergic interneurons. Infants display violent spasms, exaggerated trunk and limb rigidity and frequent tremors in response to unexpected trivial noise or touch stimuli [1]. Neonates undergoing hyperekplexia have swallowing difficulties and weaning complications and may suffer brain damage or even sudden death caused by respiratory apnea. Adults experience disabling motor alterations and recurrent unprotected falls throughout their entire life [2]. Hyperekplexia results from impaired glycinergic neurotransmission. While no causative treatment is available, current symptomatic pharmacotherapy aims to restore brainstem inhibition, primarily through the use of benzodiazepines such as clonazepam [2].

Brain stem and spinal cord glycinergic pathways control muscle tone, motor rhythms, spinal reflex responses, and sensory and nociceptive information [3,4]. Glycine released by glycinergic interneurons activates strychnine-sensitive glycine receptors (GlyRs), which permit chloride influx through the postsynaptic membrane, thus suppressing the propagation of excitatory postsynaptic potentials [4]. The neuronal glycine transporter GlyT2 is a key component of inhibitory glycinergic synapses. It removes glycine from the synaptic cleft through active Na^+^, Cl^−^ and glycine cotransport and contributes to the termination of the glycinergic signal together with its glial counterpart GlyT1 [5]. GlyT2 activity supplies substrate to the low-affinity vesicular glycine transporter VIAAT and hence allows the maintenance of the synaptic vesicle content. The deletion of the GlyT2 gene in mice reproduces the symptoms of human hyperekplexia [6], as do loss-of-function mutations in the GlyR [7]. Sequencing genomic DNA from patients with startle disease identified mutations in the human GlyT2 gene (*SLC6A5*) as the cause of about 25% of known hyperekplexia cases [8,9,10]. Patients with GlyT2 gene mutations are significantly more prone to have recurrent infantile apneas and developmental delay than those affecting the α1 subunit of the GlyR gene *GLRA1*, which are the majority of hyperekplexia-causing mutations (60%) [11].

GlyT2 belongs to the SLC6 family of neurotransmitter transporters, which also comprises transporters for GABA and monoamines [12]. Structurally, these proteins have cytoplasmic N- and C- termini and twelve transmembrane domains (TMs) arranged into two topologically inverted repeats connected by external and internal loops [13]. During the transport cycle, the substrate and ions are exposed alternately to each side of the membrane as the protein structure adopts outward, occluded or inwardly directed conformational states. Models of the SLC6 transporter structure were first based in the prokaryote ortholog LeuTAa [13] and later confirmed by data from eukaryote crystals and cryo-electron microscopy [14,15,16,17].

The synthesis of GlyT2, like other plasma membrane proteins, takes place in ribosomes associated with the endoplasmic reticulum (ER) and, as the polypeptide chain emerges, it is co-translationally translocated to the ER membrane. Protein folding and modification take place, assisted by ER chaperones, such as calnexin (CNX), while intracellular N- and C-termini lie in the cytoplasm [18]. Properly folded proteins are transported from the ER to the Golgi apparatus in vesicles coated by coatomer protein II (COPII) by using the Sec24D adaptor or, if permanently unfolded, they are exported for ER-associated degradation (ERAD) [19]. Several hyperekplexia variants prevent transporter trafficking to the plasma membrane [20,21]. In this group, there are some GlyT2 mutants whose folding can be facilitated by using chemical chaperones such as 4-phenylbutyric acid (PBA) or N-arachidonoyl glycine (NAG) [22].

In this report, we characterize a new GlyT2 variant G449E originally identified in an infant with startle disease homozygous for the DNA substitution c.1346G>A. The variant is inherited in a recessive form and our study proves that the mutation is a loss of function, causing a reduced expression of a defective transporter unable to produce mature protein and glycine transport. We demonstrate that the mutant transporter tenaciously targets lipid rafts, although it does not display a negative interaction with wild-type trafficking due to efficient degradation. Mutant expression alters cell proteostasis through triggering the unfolded protein response (UPR) and interferes with lipid raft-dependent processes, as monitored by proteomics. Learning the causes of in the activity of mutant transporters and their effects on glycine-mediated neurotransmission can guide future research with therapeutic relevance.

## 2. Results

### 2.1. Patient Information

A new GlyT2 variant has been localized in an infantile patient from the United Kingdom. The patient is a little boy, the second child to first cousin parents, who has stiffening episodes and has been diagnosed with hyperekplexia. The patient’s older sister is well, and there is no relevant wider family history. His medical history can be summarized as follows. Antenatal movements were increased with a pulsating quality. He was born by normal delivery at term and was noted to have a prominent startle reflex. He was jittery in the newborn period; this progressed to episodes of rhythmic symmetrical stiffening, lasting for a few seconds to a few minutes. Investigations for seizures were normal. An MRI scan of his brain was normal, and EEG showed background activity within normal limits with no epileptiform activity. Metabolic screening was normal. He was reviewed by a neurology team at 4 weeks of age and thought to have hyperekplexia due to stiffening and jitteriness all over his body associated with crying. This was seen particularly in response to startling stimuli such as sudden noises or movement or being driven over bumpy roads or pushed over cobbles in a pram.

### 2.2. Genetic Analysis

A genomic DNA sample from the proband was scanned on all 16 coding exons and extended flanking intronic regions of *SLC6A5* (11p15.1), encoding human GlyT2. The proband was found to have a variant of unknown significance in the homozygous state in exon 8 of the *SLC6A5* gene—c.1346G>A. The sequencing of this exon in samples from the parents revealed heterozygosity for the same substitution (Supplementary Figure S1). The patient had the sequencing of *GLRA1* (5q33.1) and *GLRB* (4q32.1) with no detected abnormalities. At the level of proteins, the substitution results in a missense mutation p.Gly449Glu. The substitution has been classified as “probably damaging” with a score of 1.000, the highest, in the PolyPhen-2 program [23].

**Figure 1:**
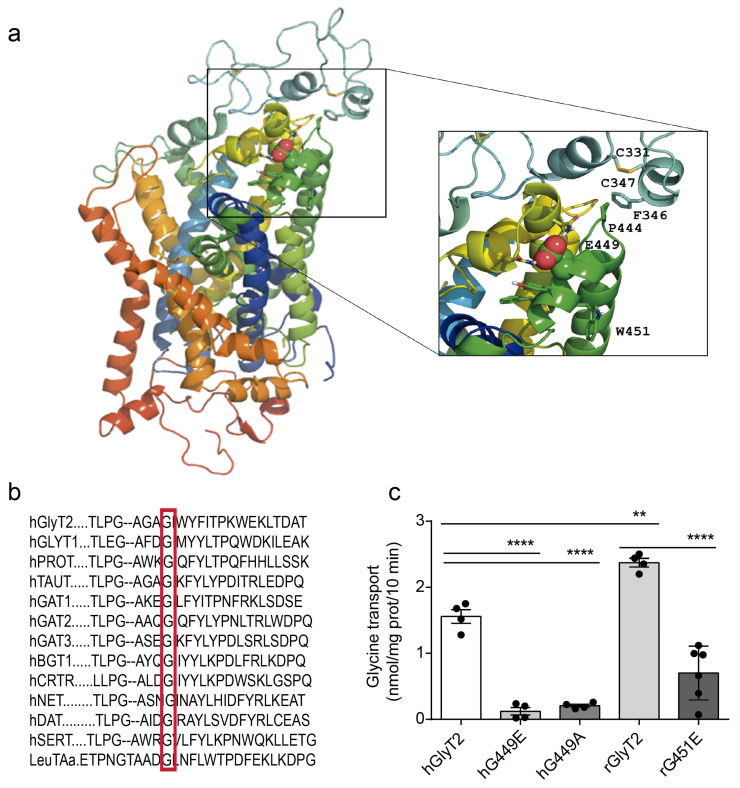
Structural and functional analysis of the G449E variant of human GlyT2. (**a**) A three-dimensional atomic model in a rainbow-colored ribbon representation displaying Glu449 atoms as spheres. The image is a representative structure taken after 1250 ns of unrestrained molecular dynamics simulation in a box of water. Note that the N- and C-termini are both located at the bottom whereas ECL3 is located on the other side of the transmembrane helices. Residues relevant to the discussion are labeled in the enlarged, framed area on the right. (**b**) The sequence alignment of the GlyT2 EL3 region in human SLC6 family members. Sequences were obtained from the NCBI (www.ncbi.nlm.nih.gov, accessed on 20 April 2023) and were aligned using ClustalW 2.1 software (European Bioinformatics Institute, Hinxton, UK). Conserved glycines are indicated by a red box. (**c**) COS7 cells transiently expressing the indicated GlyT2 transporter from a human (h) or rat (r) were tested for [^3^H]glycine transport. **** *p* < 0.0001, and ** *p* < 0.01 indicate significant difference from the wild-type in a one-way ANOVA with Sidak’s multiple comparison test (n = 4 independent cell culture preparations).

The new variant introduces glutamate instead of glycine at position 449. The homology modeling of GlyT2 using the crystal structure of the dopamine transporter (DAT) from Drosophila melanogaster [PDB code 4M48, [24]] located Gly-449 at the third external loop (EL3) of the human glycine transporter GlyT2 (Figure 1a). Phylogenetic comparisons of the EL3 region of GlyT2 show the high evolutionary conservation of Gly-449 among the phylogenetic scale, and the alignment of GlyT2 with other Na^+^/Cl^−^-dependent neurotransmitter transporters of the SLC6 family demonstrates that glycine is found at the equivalent position in all human neurotransmitter transporters (Figure 1b). This suggests that Gly-449 is necessary for transporter function.

### 2.3. Mutagenesis and Functional Characterization

We generated the recombinant human GlyT2 variant in a eukaryotic expression vector and the features of the transporter were evaluated upon heterologous expression. G449E showed transport levels undistinguishable from mock-transfected cells, indicating that the transporter variant is inactive (Figure 1c). The substitution of Gly-449 by alanine (instead of glutamate) also generated an inactive transporter, confirming that Gly-449 is necessary for transporter structure and/or activity. In addition to the mutation in the human GlyT2, we generated the equivalent mutation in the rat GlyT2 cDNA, which was numbered rG451E, since, in the rat sequence, there were two additional amino acids in the GlyT2 N-terminus. The rat mutant was equally inactive (Figure 1c).

### 2.4. Transporter Expression

**Figure 2:**
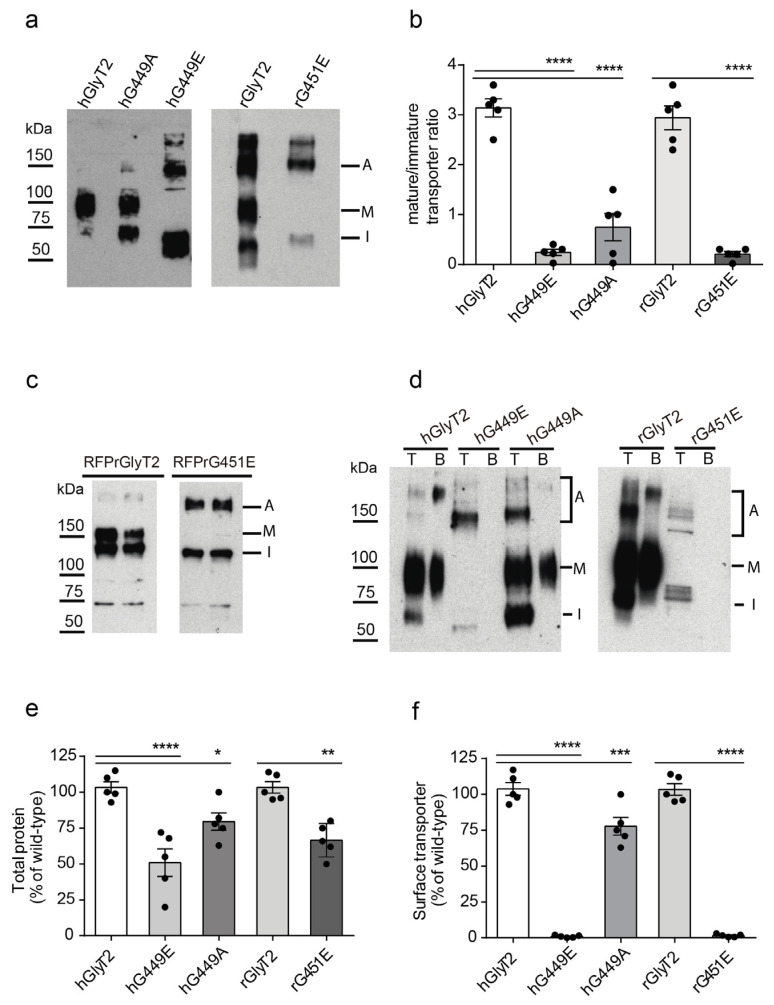
The mutant transporter is not expressed at the plasma membrane. COS7 cells transiently expressing the indicated transporters were subjected to a Western blot (**a**–**c**). A, aggregate; M, mature transporter band; I, immature transporter band. (**b**) The quantification of the ratio of mature to immature transporters in (**a**). (**c**) RFP-tagged transporter constructs. (**d**) The surface biotinylation of the indicated transporters. T, total transporter; B, biotinylated transporter. (**e**) The normalized total expression of transporters. (**f**) Normalized surface-resident transporters. **** *p* < 0.0001, *** *p* < 0.001, ** *p* < 0.01, and * *p* < 0.05 in a one-way ANOVA with Sidak’s multiple comparison test (n = 6 independent cell culture preparations).

A Western blot analysis of GlyT2 expressed in COS7 cells depicts a 100 kDa mature form, an immature form of 75 kDa, with some aggregates of both running as higher size bands (Figure 2) [18,25]. Sometimes, several proteolytic fragments are also observed. Figure 2 shows that, in contrast to wild-type GlyT2, the new GlyT2 variant is apparently composed by a single form corresponding to the immature transporter together with aggregates of it (Figure 2a,c,d). By using very high Western blot film exposures, we calculated the ratio of mature to immature transporters and found it was more than 3 times reduced for the glutamate substitutions and about half for the alanine substitution, suggesting transporter maturation is somehow altered in the mutants (Figure 2a,b). The tendency of the variant protein to generate large-size aggregates together with the almost complete absence of the mature form was especially obvious in the RFP-tagged transporters we constructed for other uses (Figure 2c).

Since the mature 100 kDa transporter is the unique form of GlyT2 that can reach the plasma membrane [18], it was expected that the mutant would not be expressed at the cell surface. In fact, this behavior of the mutant was assessed by surface biotinylation (Figure 2d–f). Substitutions to the glutamate of the human GlyT2 (hG449E) and the rat GlyT2 (rG451E) could not be surface-biotinylated. In contrast, the mutant bearing alanine instead of glycine (hG449A) was able to reach the plasma membrane, despite being inactive as G449E (Figure 1).

**Figure 3:**
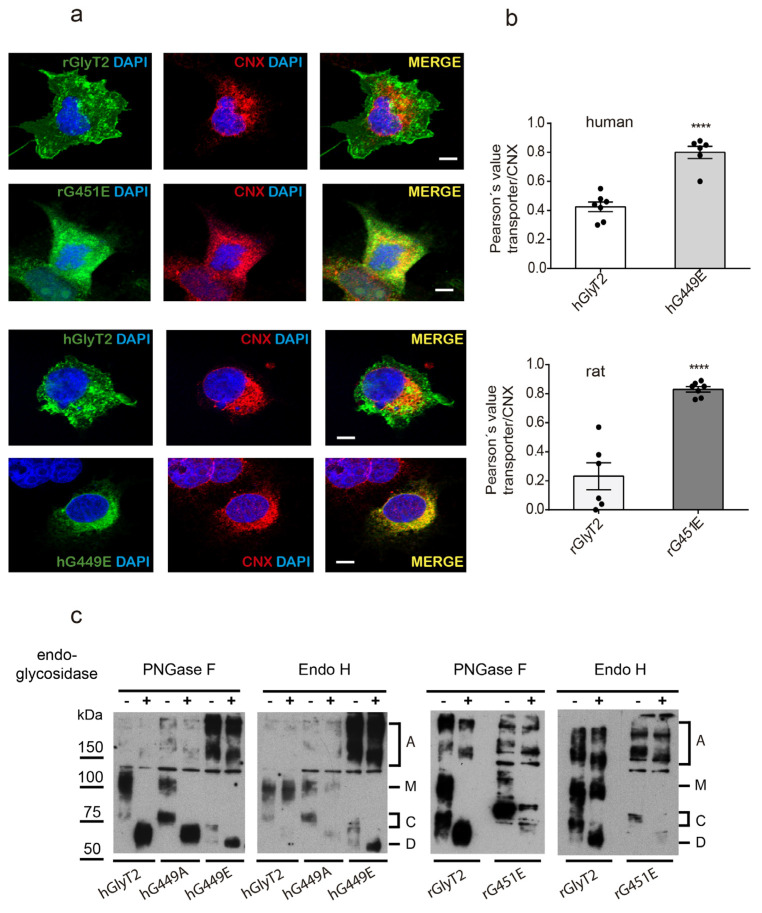
The transporter variant is retained in the ER. (**a**) COS7 cells expressing hGlyT2, hG449E, rGlyT2 or rG451E were immunolabeled for the indicated transporter and the ER chaperone CNX. Transporters are shown in green, CNX is shown in red and DAPI is shown in blue (bars: 10 μm). (**b**) The quantification of the colocalization between the transporters and CNX using Pearson’s value of correlation. **** *p* < 0.0001 using the T comparison test (n = 50 counted cells). (**c**) The carbohydrate modification of the transporter variant. Lysates of COS7 cells expressing the indicated transporters were treated overnight with the vehicle alone (endoglycosidase buffer, −) or with the indicated endoglycosidase (+) in denaturing conditions and then resolved by SDS-PAGE as described in the Material and Methods Section 4 (3 experiments were conducted with the same results). A, aggregate; M, mature glycosylated transporter; C, core glycosylated transporter; D, de-glycosylated transporter.

Furthermore, dual immunofluorescence analysis revealed glutamate substitution transporter mutants displayed increased colocalization levels with the endoplasmic reticulum (ER) marker calnexin (CNX) compared to the wild-type in both human and rat transporters (Figure 3a,b). This strongly suggests that the mutant is retained in the secretory pathway. As confirmation, we assessed the glycosylation state of the human transporter variant by performing digestions with endoglycosidases. PNGase F removes GlyT2 N-linked glycans from both the 75 and 100 kDa forms, yielding a 60 kDa band that corresponds to the non-glycosylated protein core. The sensitivity of the mutants to this enzyme indicates they have received the initial 14-sugar chain from dolichol and are immaturely glycosylated proteins. By contrast, Endo H cleaves high-mannose N-linked oligosaccharides, allowing an assessment of Golgi-mediated glycosylation [26]. ER-retained proteins are Endo H-sensitive, while mature proteins that pass through the Golgi become Endo H-resistant. The 100 kDa mature GlyT2 form is Endo H-resistant, which corresponds to a glycoprotein that exited the ER. The transporter variant, as the immature 75 kDa band, was sensitive to Endo H, indicating that it is a protein retained in the ER that exhibits only incomplete glycosylation [19] (Figure 3c). These data indicate that the maturation of the transporter through the secretory pathway is arrested by the introduction of the charged and voluminous glutamate at position G449, whereas the alanine substitution permits transporter maturation and plasma membrane delivery, although not transport activity.

### 2.5. Lipid Raft Expression

**Figure 4:**
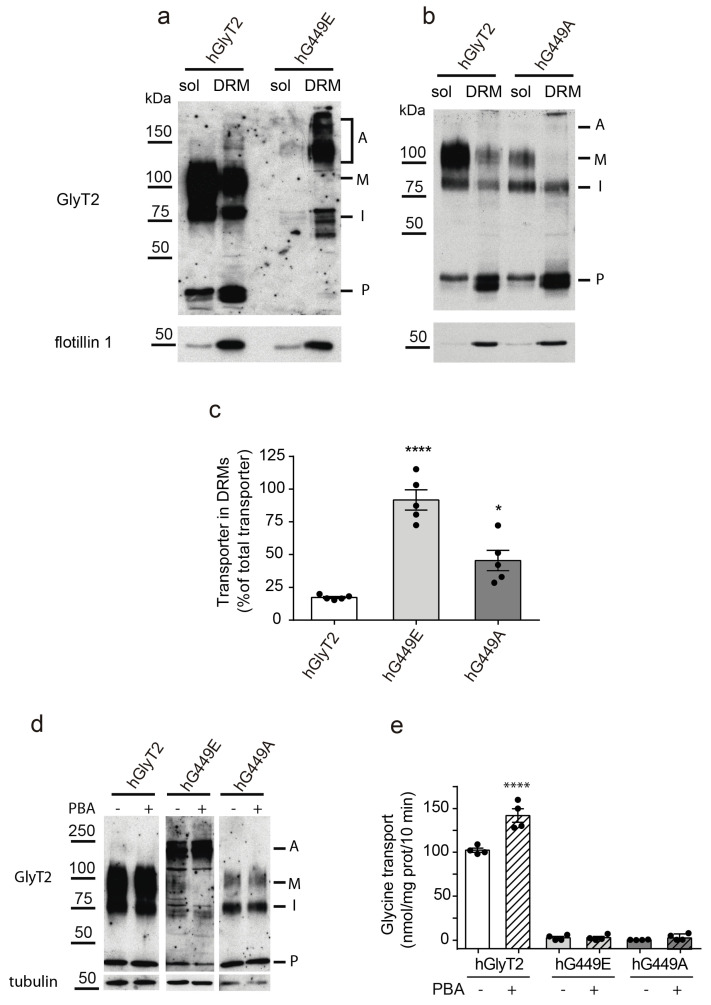
The mutant transporter has an aberrant inclusion in lipid raft membranes. (**a**,**b**) Detergent-resistant membranes (DRMs) were isolated from COS7 cells expressing hGlyT2 (**a**,**b**) or hG449E (**a**) or hG449A (**b**), as described in the Material and Methods Section 4, and subjected to a Western blot for GlyT2 detection and the detection of the DRM marker flotillin 1. Sol, soluble fraction; DRM, detergent-resistant membranes. A, aggregate; M, mature transporter; I, immature transporter; P, proteolytic fragment. (**c**) The quantification of the percentage of GlyT2 immunoreactivity present in DRMs normalized by total GlyT2 immunoreactivity. **** *p* < 0.0001 and * *p* < 0.05 in a one-way ANOVA with Sidak’s multiple comparison test (n = 4 independent cell culture preparations). (**d**) COS7 cells transiently expressing the indicated transporters were treated with 1 mM 4-phenylbutyric acid (PBA, +) or vehicle (−) for 48 h, as described in [22]. Then, cells were subjected to a Western blot to detect GlyT2 and tubulin as a loading control (**d**) or to a [^3^H]glycine transport assay (**e**). **** *p* < 0.0001 in a one-way ANOVA with Sidak’s multiple comparison test (n = 3 independent cell culture preparations).

The propensity to generate high-order aggregates exhibited by the new transporter variant, as shown in Figure 2, may sometimes occur when the protein has increased hydrophobicity. This could be caused by the alteration of the glycosylation pattern illustrated in Figure 3c. This condition may have consequences in the ability of the mutant to be included in lipid rafts. Lipid rafts, also called detergent-resistant membranes (DRMs) using an operational definition, are membrane subdomains highly enriched in cholesterol and sphingolipids that compartmentalize cellular processes [27,28]. GlyT2 displays optimal transport activity when it is associated with these subdomains at the cell surface, in which most of the transporter resides both in rat brainstem primary neurons and synaptosomes. These domains are platforms where several regulatory mechanisms control GlyT2 function [29]. We evaluated the capability of the raft association of the hG449E mutant by isolating DRMs from cells individually expressing the wild-type or the mutant transporter (Figure 4a,c). Whereas the wild-type transporter was distributed between the soluble fraction and the DRMs so that about 13% of the total protein was present in DRMs, as previously quantified in other systems [29], almost the whole amount of the transporter variant was associated with these domains. The alanine substitution mutant also shows an increased tendency to be included in lipid rafts, although much lower than that of G449E (Figure 4b,c). Constitutive association with lipid rafts has been observed for mutants of other membrane proteins [30]. The increased propensity of the human variant to be included in DRMs, despite being a folding-defective mutant, suggests an aberrant insertion of the mutant transporter in the lipid raft membranes. We tried to relieve the folding defect and therefore the maturation arrest of the mutant transporter using an effective chemical chaperone: 4-phenyl butyric acid (PBA). This compound is able to facilitate the biogenesis of the wild-type transporter and several hyperekplexia mutants, as we proved previously [19,22]. However, in contrast to the wild-type transporter, the human variant could not be rescued according to an abnormal insertion in ordered membrane subdomains (Figure 4d,e).

**Figure 5:**
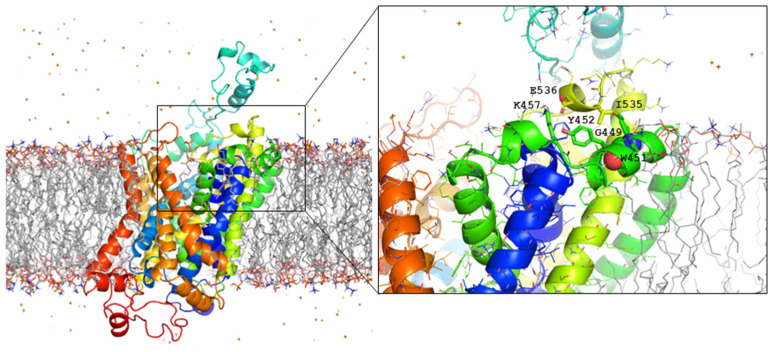
A side view of the wild-type human GlyT2 embedded in a lipid bilayer (wireframe). Rainbow-colored ribbon representation. The cytosolic side is at the bottom and the extracellular space is at the top. Water molecules have been omitted for clarity, but the counterions are displayed as stars. The enlarged region shows the location of Gly449 and surrounding residues relative to the membrane, whose interactions both among themselves and with the polar heads of the vicinal cholesterol and phospholipid molecules in the outer leaflet are likely to change as a result of the G449E replacement.

Our computational models of human GlyT2 locate Gly449/Glu449 on helix H17, which is found close to the ECL3 region (Figure 1a) and also in contact with the polar heads of the phospholipids that make up the outer layer of the membrane (Figure 5). Upon refinement, the Glu449 carboxylate reorientates slightly to avoid steric clashes with Ile535 and establishes hydrogen bonding interactions with the sidechain carboxamides of Asn529 and Asn534 (Figure 1a). Of particular interest is the vicinal Trp451, whose indole ring is exposed outwards, thereby providing a suitable platform for cation–π interactions [31] with the trimethylammonium head of phosphocholine-containing phospholipids (or sphingolipids, in the case of lipid rafts [32]). Furthermore, this short helix contains a contiguous WYF motif that may be restructured to enhance this type of biologically important interaction and possibly (i) give rise to a misfolded protein that cannot escape from the ER [33,34] and (ii) promote the formation of large-size aggregates. Although it cannot be ruled out that the multimerization of the native G449E variant may arise from experimental manipulation, the enhanced aggregation tendency of this variant relative to the wild-type transporter supports this hypothesis. In addition, the ECL3 region is very close to residues that have been related to dimer formation in several GlyT2 [35] and GlyT1 [36] constructs treated with crosslinkers.

### 2.6. Wild-Type–Mutant Coexpression

**Figure 6:**
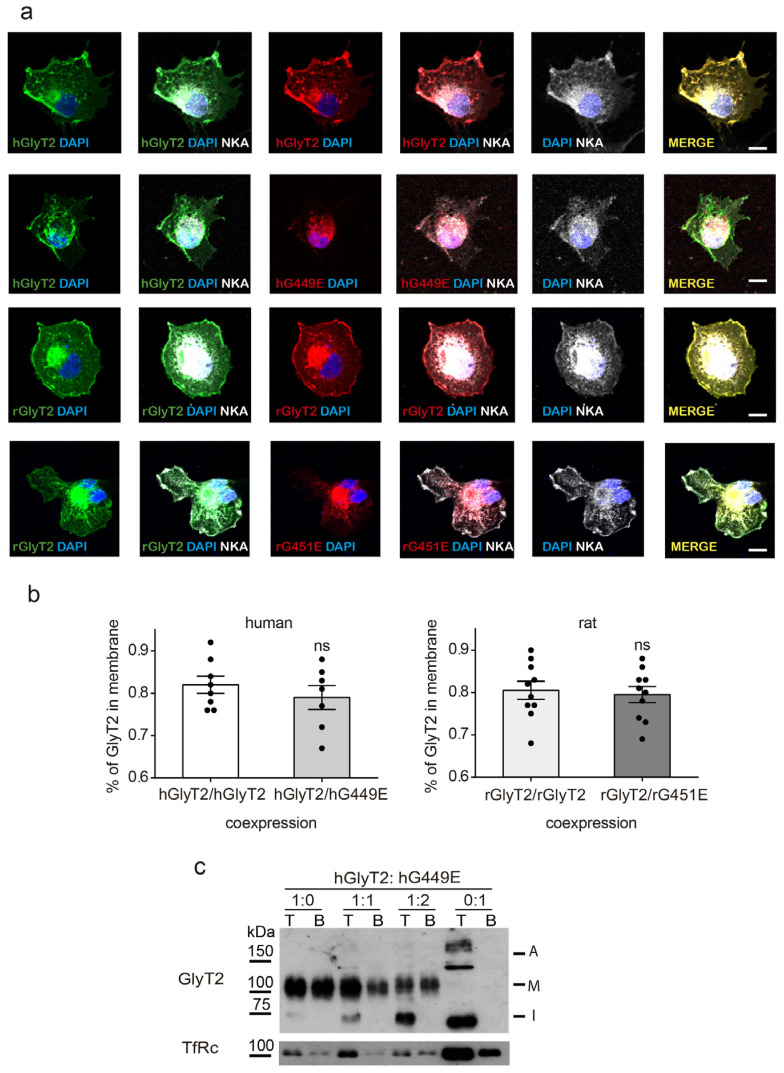
Wild-type surface expression is not altered by mutant coexpression. (**a**) COS7 cells transfected 48 h before were immunolabeled for expressed transporters, the plasma membrane marker NKATPase (gray) and the nucleus marker DAPI (blue). The transporters expressed for each condition were GFP-hGlyT2 and Flag-hGlyT2 in the first row, GFP-hGlyT2 and HA-hG449E in the second row, GFP-rGlyT2 and RFP-rGlyT2 in the third row, and GFP-rGlyT2 and RFP-rG451E in the fourth row (bars: 10 μm). Three-channel confocal images were obtained (green or red for transporters and gray for NKATPase), and the regions occupied by NKATPase at the external areas of the cell were considered the plasma membrane when using the ImageJ 1.54k (National Institutes of Health, Bethesda, MD, USA) ROI manager tool. After applying an automatic threshold for adjustment, fluorescence intensity was measured separately for membrane and intracellular regions, and the proportion of the transporter at the plasma membrane was calculated (**b**). This process was performed in at least 30 cells/condition. ns, non-statistically significant in an unpaired *t* test. (**c**) COS7 cells expressing hGlyT2 alone (1:0) or hG449E alone (0:1) or hGlyT2 together with the hG449E mutant in a ratio of hGlyT2/hG449E cDNA 1:1 or 1:2, were subjected to surface biotinylation and a Western blot. T, total protein (3 µg); B, biotinylated protein (9 µg). For hG449E, these amounts were multiplied by 3. Transferrin receptor (TfRc) immunoreactivity was used as a loading control. A, aggregate; M, mature transporter band; I, immature transporter band.

The anomalous insertion of the mutant transporter in the membrane led us to question whether the wild-type traffic was altered by the joint coexpression of the mutant. This condition could take place in heterozygous individuals, and although we are not aware of hyperekplexia-associated symptoms of the heterozygous parents of the proband, sometimes, the manifestations are subtle and may not be perceived. Therefore, we constructed differentially tagged wild-type and mutant transporters and coexpressed them in COS7 cells. Co-transfected cells were analyzed after multiple immunofluorescence and the influence of the mutant on wild-type GlyT2 plasma membrane expression was assessed by colocalization with a plasma membrane marker (Figure 6a,b).

The percentage of tagged GlyT2 present at the surface was the same whenever it was coexpressed with the differentially tagged mutant or with the wild-type, both in human and rat transporters (Figure 6b). In addition, the increased expression of the untagged variant together with the untagged wild-type did not decrease the amount of the surface-biotinylated wild-type transporter, as monitored by surface biotinylation and a Western blot (Figure 6c).

One possible explanation for the wild-type surface expression remaining unchanged despite its simultaneous expression with a mutant that exhibits aberrant interaction with the membrane is the absence of a protein–protein interaction between the wild-type and mutant proteins. Oligomer formation is a requirement for ER export by the SLC6 transporters [37]. It was proven by ours that several other GlyT2 hyperekplexia mutant proteins oligomerize. In fact, the pathogenic mechanism of the two dominant mutations found in hyperekplexia patients relies on the formation of heteroligomers carrying wild-type and mutant protomers [19,25]. To know whether the mutant under study was able to oligomerize with the wild-type, we coexpressed differentially tagged wild-type and mutant transporters and perform co-immunoprecipitation experiments (Supplementary Figure S2). Immunoprecipitation using a one tag-directed antibody (α-myc) permitted the detection of the second tag-labeled transporter (α-flag) in the precipitated complex. This indicates wild-type and mutant transporters indeed do interact.

### 2.7. Mutant Transporter Degradation

Another mechanism that may protect wild-type transporter surface expression in the presence of the mutant is the efficient degradation of the mutant protein.

**Figure 7:**
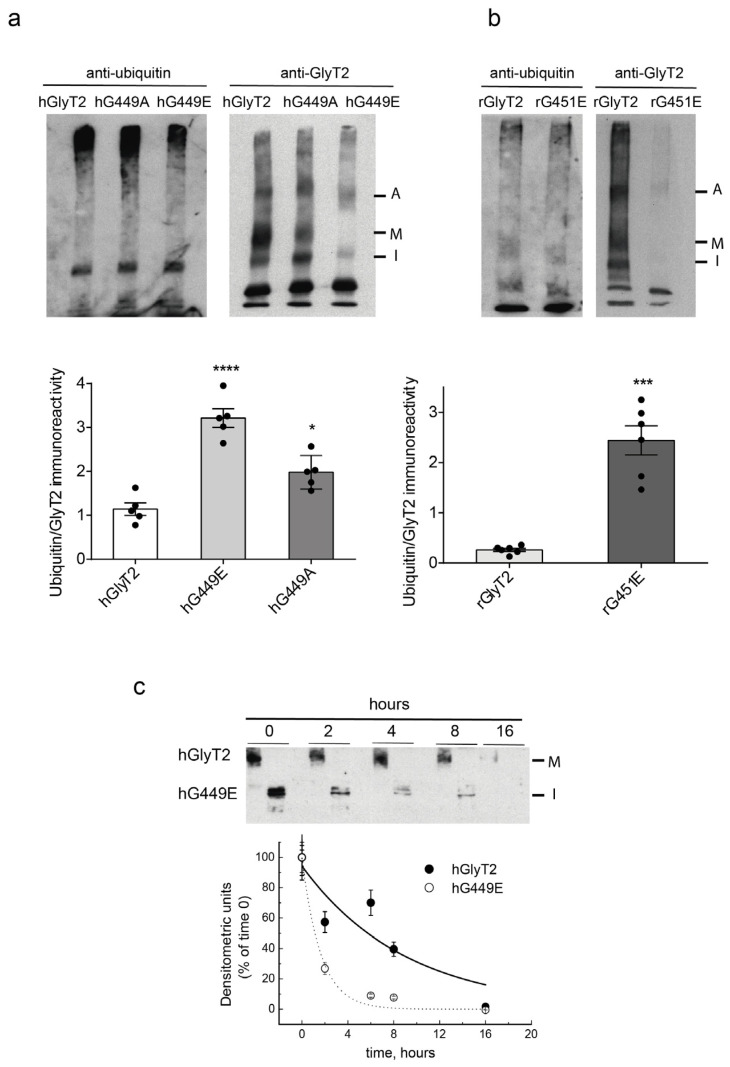
The ubiquitination levels and half-life of G449E. (**a**,**b**) Lysates of COS7 cells transiently expressing the indicated transporters were subjected to immunoprecipitation against GlyT2 in high SDS, as described in the Material and Methods Section 4.11, and the ubiquitination of the transporter was determined by immunoblotting against ubiquitin. The quantification of transporter ubiquitination is shown in the bar diagrams. Blots were probed against GlyT2 to normalize the ubiquitination signal against the amount of GlyT2 immunoprecipitated in each case. **** *p* < 0.0001, *** *p* < 0.001 and * *p* < 0.05 using a one-way ANOVA with Dunnett’s multiple comparison test (n = 6 independent cell culture preparations). (**c**) COS7 cells expressing hGlyT2 or hG449E were treated with 25 μM cycloheximide to block protein synthesis, and the transporter was monitored by immunoblotting cell lysates with an anti-GlyT2 antibody at 0, 2, 4, 8 and 16 h (3 independent experiments were conducted with the same results). A, aggregate; M, mature transporter band; I, immature transporter band.

Since the synthesis of GlyT2 involves ER-associated degradation (ERAD), and also its intracellular trafficking is regulated by ubiquitination [19,38,39], we first examined the ubiquitination ability of the mutants by the immunoprecipitation of the transporters in stringent conditions followed by immunodetection with anti-ubiquitin antibodies (Figure 7a,b). Mutant transporters exhibited a higher degree of ubiquitination compared to the wild-type, with the level of modification positively correlating with the severity of the mutant’s damaging effect. For the human glutamate substitution variant, a threefold increase in ubiquitination was detected, while the alanine mutation showed a twofold increase. Interestingly, this correlates with a threefold reduction in the half-life of the human GlyT2 variant (1 h for hG449E compared to 3 h for the wild-type), suggesting that it is a folding-defective mutant that undergoes degradation through ERAD (Figure 7c). The combined expression of the wild-type transporter with the variant does not appear to increase the half-life of the wild-type, unlike what is observed in other loss-of-function mutants of different transporters, such as CFTR in cystic fibrosis [40].

### 2.8. Proteomic Analysis

The mutant transporter, displaying a large insertion in lipid rafts, may have disturbing actions in the cells. Therefore, we revisited the possible role of the new variant, which remained arrested at the ER, as a cell proteostasis disturber. For this purpose, we performed quantitative proteomics to analyze the differential abundance of proteins between the cells expressing the mutant and those expressing the wild-type. A total of 5106 proteins were quantitated (Supplementary Table S1).

**Figure 8:**
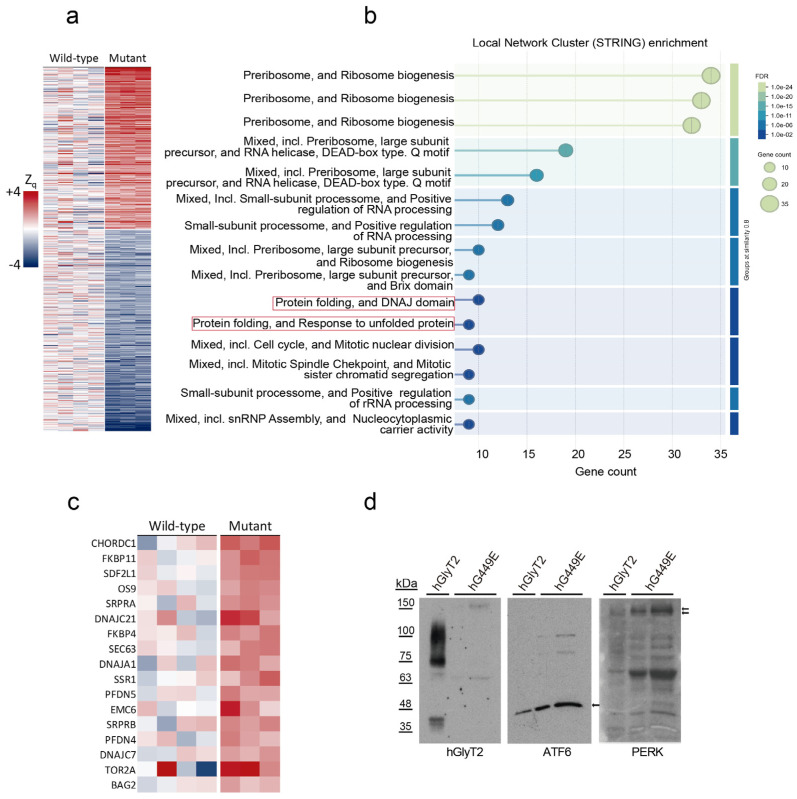
Comparative proteomics of cells expressing hG449E and hGlyT2. (**a**) Heatmap showing the proteins more abundant or reduced in cells expressing the hG449E mutant (n = 4) when compared to those expressing the wild-type transporter (n = 3). Data show the proteins with an average difference in Zq values between groups higher than 1.3 or lower than −1.3. (**b**) Enrichment analysis showing molecular functions increased in the cells expressing G449E mutant. (**c**) A heatmap showing proteins belonging to the UPR according to the STRING database [42]. Color code as in (**a**). (**d**) A Western blot of cells expressing hGlyT2 or hG449E for the visualization of the indicated UPR markers. Arrows indicate the relevant proteins.

Interaction network and functional category enrichment analysis of proteins that were increased in the cells expressing the hG449E mutant (Figure 8a) showed a significant enrichment of preribosome and ribosome biogenesis, translation regulation, mitotic cell cycle, protein folding and DNAJ domain, as well as unfolded protein response (UPR) (Figure 8b and Supplementary Figure S3). To further analyze the alteration in UPR, we manually selected, from the whole proteome, the proteins related to heat shock, chaperone activity, or the UPR, finding a generalized increase in all 17 proteins shown in Figure 8c. All these proteins are included in reactome pathways involving at least one of the three UPR sensors: inositol requiring enzyme 1 (IRE1), pancreatic endoplasmic reticulum kinase (PERK), and activating transcription factor 6 (ATF6) [41]. These data support the notion that the expression of the hG449E mutant triggers the UPR. In fact, we could detect increased levels of UPR markers ATF6 and phospho-PERK in the cells expressing hG449E compared to those expressing hGlyT2 (Figure 8d).

**Figure 9:**
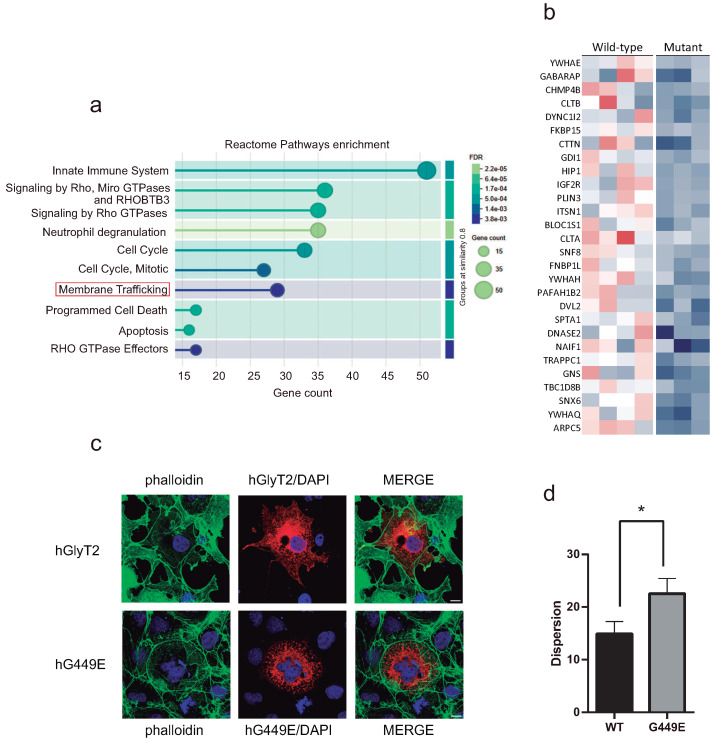
Comparative proteomics of cells expressing hG449E and hGlyT2. (**a**) Enrichment analysis showing molecular functions decreased in the cells expressing G449E mutant. (**b**) A heatmap showing proteins belonging to membrane trafficking reactome pathways according to the STRING database [42]. Color code as in Figure 8a. (**c**) COS7 cells expressing hGlyT2 or hG449E were immunolabeled for the expressed transporters (red), the nucleus marker DAPI (blue), and the actin marker phalloidin (green) (bars: 10 μm). (**d**) The quantification of F-actin fiber organization was performed using the Directionality plugin in Fiji 2.9.0 (open-source software developed by the Fiji community), as described in the Material and Methods Section 4.6. The dispersion parameter reflecting the angular spread of fiber orientations is depicted in the plot. Lower values indicate greater alignment and organization, while higher values reflect increased stress fiber disorder (44–46 cells/condition). * *p* < 0.05 in a Student’s *t* test.

The same analysis was performed on the proteins most decreased upon mutant expression (Figure 8a). Among the enriched categories, we found membrane trafficking (Figure 9a and Supplementary Figure S4). A further inspection of the 28 proteins belonging to this category revealed a generalized decrease in all of them (Figure 9b). Interestingly, among these, 20 are proteins associated with membrane rafts or interacting with rafts, 7 are not raft-related and 1 is a protein whose knock out triggers the UPR in immune cells [43]. Therefore, the expression of a mutant constitutively included in rafts promotes a decrease in membrane trafficking proteins, the majority of which are related to membrane rafts (71.4% of total).

The expression of the transporter variant did not only promote a decrease in many proteins usually associated with lipid rafts, including YWHAE [44], GABARAP [45], DYNC1I2 [45] or SNF8 [46], but also decreased proteins belonging to or interacting with the actin cortical cytoskeleton, such as CTTN [47], FNBP1L [48] and SPTA1 [44], suggesting that raft-related events are negatively affected by mutant expression. Phalloidin labeling revealed that cells expressing the variant displayed less organized actin fibers compared to the wild-type, as supported by significant differences in actin dispersion values (Figure 9c,d). This dispersion metric reflects stress fiber disorder, with lower values indicating greater alignment and organization. Additionally, mutant-expressing cells exhibited a more compact or rounded morphology, in contrast to the more extended and structured shape of wild-type-expressing cells. While these often showed fine cellular projections (filopodia or lamellipodia), these features were fewer and more diffusely distributed in cells expressing the mutant.

In summary, our data confirm that while the new hyperekplexia variant is a loss of function regarding GlyT2 activity, it may gain the function of altering proteostasis through triggering the UPR and probably interfering with lipid raft-dependent processes.

## 3. Discussion

A new GlyT2 variant identified in a hyperekplexia patient was characterized. The mutation appeared in an infant with startle disease homozygous for the DNA substitution c.1346G>A, encoding for a transporter harboring the non-conserved substitution G449E. The Gly449 of human GlyT2 is conserved among all species examined and is also found in all members of the SLC6 family. This strict conservation suggests an important role that is either structural or functional. We expressed the recombinant GlyT2 variant in heterologous cells and analyzed the pathogenic mechanism of the mutation. Our study proves the mutation is a loss of function and causes the reduced expression of a defective transporter unable to produce mature protein and glycine transport. In comparison with the wild-type, the mutant transporter is retained in the ER as proven both by increased colocalization with the ER marker calnexin and by an inspection of its carbohydrate modification. The separation of detergent-resistant membranes from cells expressing the transporters revealed that the mutant protein is more prone to being included in lipid rafts than the wild-type. This, together with the variant’s tendency to form large-size aggregates, suggests its anomalous insertion in the membrane. This feature is in good agreement with its impossibility of being rescued by chemical chaperones, which are effective on the wild-type such as phenylbutyrate. Conversely, the coexpression of the mutant and wild-type did not apparently affect the trafficking of GlyT2 since its surface expression was not altered in the presence of the mutant. This discards any dominant negative effect on GlyT2 trafficking, in agreement with the lack of hyperekplexia symptoms of the patient’s parents. The absence of a negative interaction of the mutant with the trafficking of the wild-type could be facilitated by the efficient degradation of the mutant transporter after ubiquitination, a mechanism that may help to alleviate the observed alteration of cell proteostasis [41]. In fact, ubiquitination levels of the mutant are increased compared to those of the wild-type.

The trafficking of SLC6 transporters requires oligomerization in the ER. The ability of forming common heteromers containing wild-type and mutant transporters seems not to be lost by the mutant. However, the large presence of the transporter variant in lipid rafts, despite its absence from plasma membrane, suggests there is a separation of the wild-type and mutant at some level along the secretory pathway. Although it is clear that GlyT2 is present in lipid rafts in the plasma membrane, which is the optimal location to exert glycine transport function, the role of lipid rafts in the delivery of GlyT2 to the surface has not been studied. In brain stem synaptosomes, GlyT2 reaches the plasma membrane in membrane vesicles requiring syntaxin-1, a SNARE protein present in membrane rafts [49]. In addition, when GlyT2 is constitutively internalized from surface lipid rafts and subsequently recycled to the plasma membrane, it remains associated with rafts in subcellular recycling structures [39]. Lipid rafts in the ER are signaling platforms involved in different pathways including endoplasmic reticulum associated-degradation (ERAD), which involves retrograde transport out of the ER assisted by ER and cytosol chaperones, and the cytosolic ubiquitin and 26S proteasome protein degradation system [50]. A major feature of raft domains is to segregate specific elements with the aim of regulating their interactions with other membrane components, i.e., lipids and proteins, and hence their activity. One point where the transporter variant and the wild-type can be segregated is at the level of ER lipid rafts from which the mutant is directed to retrotranslocation, ubiquitin labeling and degradation through ERAD. Although proteasome-associated degradation is also involved in GlyT2 synthesis as it is in many plasma membrane proteins [18], the mutant displays an increased use of this pathway that reduces its half-life. This, together with the mutant’s large insertion in lipid rafts, may cause disturbing actions in ER. It may interfere with calcium load in the ER through mitochondrial associated membranes (MAMs), which are lipid raft regions, ERLIN-mediated cholesterol synthesis, and the initiation of autophagy, among other processes triggered from lipid rafts [51,52]. Disturbances in the ER can generate a stress condition that is detected by three membrane-bound proteins acting as sensors—inositol-requiring enzyme 1 (IRE1), pancreatic ER kinase (PERK), and activating transcription factor 6 (ATF6). These proteins initiate signaling cascades that lead to the activation of the UPR to restore cellular homeostasis [53]. Our proteomic study found that the expression of the new GlyT2 variant promotes the enhanced expression of several proteins associated with these signaling pathways, suggesting that the sole expression of the hG449E mutant can trigger the UPR.

In addition, there is a dynamic interaction between lipid rafts and the underlying cytoskeleton that may regulate many facets of the function of eukaryotic cells [53]. There seems to exist a reciprocal regulation between lipid rafts and the underlying actin cytoskeleton [54]. Cortical actin plays an active part in the maintenance and remodeling of lipid rafts [55], and conversely, the composition of the membrane raft fraction may condition the association of actin filaments with membrane rafts [56]. The enhanced insertion of the mutant transporter into lipid rafts may interfere with actin-dependent processes like endocytosis and exocytosis, which are vital for membrane trafficking. In this study, phalloidin staining revealed alterations in actin cytoskeleton organization in mutant-expressing cells. Further investigation using live-cell actin markers will be essential to clarify the molecular processes underlying this disturbance caused by the novel variant. In summary, our data prove that the new hyperekplexia variant, although it is a loss of function regarding GlyT2 function, it is a gain of function through triggering the UPR and interfering with lipid raft-dependent processes.

## 4. Materials and Methods

### 4.1. Molecular Genetic Analysis of Human GlyT2 Gene (SLC6A5)

Patient samples were obtained in accordance with the Declaration of Helsinki, following ethics approval and informed consent as indicated below. Patient genomic DNA was amplified as described [25]. No abnormalities were observed in the sequences of *GLRA1* and *GLRB*.

### 4.2. In Silico Analysis and Molecular Modeling

The conservation of the mutated residues was assessed by the alignment of orthologous and human protein sequences using ClustalW software [57]. The putatively damaging effects of the predicted amino acid substitution was assessed using the PolyPhen-2 2.2.2 (Harvard University, Boston, MA, USA) program [23] that gives the results as “benign”, “possibly damaging”, “probably damaging”, or “unknown”.

Five fully atomic models of wild-type human GlyT2 and its pathogenic G499E variant were generated by the AlphaFold 3.0 (Alphabet Inc., London, UK) server [58]. The structural analysis was carried out with PDBsum1 (European Bioinformatics Institute, Hinxton, UK) [59] for additional validation and to produce diagrams, such as that shown in Supplementary Figure S5, that facilitate secondary structure assignments and nomenclature. Disulfide linkages were defined between Cys311-Cys320, and Cys331-Cys347 pairs in the third extracellular loop (ECL3). The very low root mean deviations within each set of five models (0.12–0.16 Å over 513–534 Cα atoms) and between the wild-type and the pathogenic G449E variant (0.18–0.25 Å over 461–481 Cα atoms) are in line with the current lack of sensitivity of this revolutionary methodology to single amino acid replacements that are known to have a large impact on protein stability and function [60]. For this reason, the G499E model was immersed in a cubic box of TIP3P water molecules (plus counterions) and simulated using molecular dynamics (MD).

The CHARMM-GUI 3.8 (Lehight University, Bethlehem, PA, USA) Membrane Builder pipeline [61] and the PPM 2.0 (University of Michigan, Ann Arbor, MI, USA) server [62] were employed to embed the wild-type hGlyT2 model protein in a lipid bilayer made up of cholesterol and phospholipids containing palmitic acid and a variety of polar heads (phosphatidic acid, phosphatidylethanolamine, phosphatidylcholine and phosphatidylserine) attached to the glycerol moiety as exploratory probes. Both sides of the membrane were then solvated along the Z axis with a 10 Å thick layer of TIP3P water and the bulk ion concentration was set at 0.15 M KCl. The AMBER ff14SB (University of California San Francisco, San Francisco, CA, USA) and Lipid21 (University of California San Francisco) [63] force fields were used for protein and lipids, respectively.

These initial solvated models, either free in solution (G449E) or embedded in the membrane (WT), were refined using energy minimization until the root mean square of the Cartesian elements of the gradient was less than 0.1 kcal·mol 1·Å 1. Thereafter, the systems were heated up to 303 K using the Langevin thermostat and maintained at this temperature during 5 ns to equilibrate the box dimensions and density at a constant pressure of 1 atm by means of an anisotropic Berendsen weak-coupling barostat, essentially as described previously for similar ensembles [64]. The unrestrained MD simulations proceeded under periodic boundary conditions using the pmemd.cuda code implemented in AMBER18 (University of California San Francisco), running on single NVIDIA GPUs (Nvidia Corporation, Santa Clara, CA, USA) up to a total time of 1250 ns for G449E and 300 ns for the membrane-embedded WT GlyT2.

### 4.3. GlyT2 Mutagenesis and Transporter Expression

Substitution mutants were generated with the QuikChange II Site-Directed Mutagenesis kit (Agilent Technologies, Santa Clara, CA, USA Cat#: 200519), using rat GlyT2 (rGlyT2, [65]) or human GlyT2 (hGlyT2, [66]) subcloned in pCDNA3 [67]. RFP-tagged and HA-tagged rat transporters were constructed as described elsewhere [19]. Myc-tagged and Flag-tagged human transporters were donated by F. Zafra (CBM-UAM). The complete coding region of all constructs was sequenced to verify that only the desired mutation had been introduced. Plasmids from three independent *Escherichia coli* colonies were expressed in eukaryotic cells as indicated below, and [^3^H]glycine transport and/or immunodetection was performed for verification. COS7 cells (American Type Culture Collection, Manassas, VA, USA, RRID: CVCL_0224) were used. The COS7 cell line is not listed as a commonly misidentified cell line by the International Cell Line Authentication Committee (ICLAC; http://iclac.org/databases/cross-contaminations (accessed on 7 July 2025)). Cells were expanded and refrozen during the first passage. Aliquots were thawed and used below 30 passages. Cells were grown and transfected using TurboFect Transfection Reagent (Thermo Fisher Scientific, Waltham, MA, USA, Cat#: R0532) or PEI MAX^®^ (Polyscience, Niles, IL, USA, Catalog# 24765), following the manufacturer’s protocol (2 µL reagent/µg of DNA). Cells were incubated for 48 h at 37 °C until used [68].

### 4.4. Transport Assays

Glycine transport assays in COS7 cells were performed at 37 °C in phosphate-buffered saline (137 mM NaCl, 2.7 mM KCl, 10 mM Na_2_HPO_4_ and 1.8 mM KH_2_PO_4_, pH 7.4, PBS) containing 10 mM glucose and 2 μCi/mL 2-[^3^H]-labeled glycine (1.6 TBq/mmol; PerkinElmer Life Sciences, Downers Grove, IL, USA), diluted to a final concentration of 10 μM, as described previously [67]. The reactions were terminated after 10 min (or the desired time) by aspiration, and the cells were washed and then dissolved in NaOH. The protein concentration was determined in aliquots taken from each well (Bradford), and the uptake of 2-[^3^H]glycine was measured by liquid scintillation (liquid scintillation, Opti-Fluor, PerkinElmer, LKB 1219 Rackbeta). Transport was quantified by subtracting the glycine accumulated in mock-transfected COS7 cells (cells transfected with the empty pcDNA3 plasmid) from that of the transporter-transfected cells and normalized by the protein concentration. Assays were performed in triplicate or quadruplicate.

### 4.5. Surface Biotinylation

COS7 cells expressing the transporters were labeled with PBS containing 1.0 mg/mL EZ-Link^TM^ Sulfo-NHS-Biotin (Cat#: 21217, Thermo Fisher Scientific) at 4 °C for 30 min, as described [22]. After free biotin quenching with 100 mM L-lysine in PBS, the cells were scrapped, and protein concentration was determined (Bradford). Equal amounts of proteins were lysed with RIPA buffer (1% TritonTM X-100, 0.1% SDS, 0.5% sodium deoxycholate, Tris-HCl 50 mM, NaCl 150 mM, 1 mM EDTA, 1 mM PMSF and 1:200 protease inhibitor cocktail (PI), from Sigma-Aldrich, Burlington, MA, USA, Cat# P7626 and P8465) during 30 min at 4 °C. An aliquot of the lysate was saved (total protein), and the remainder was incubated with 50% streptavidin-agarose beads (Sigma-Aldrich, Catalog# S1638) for 90 min at RT and centrifuged. Beads were washed 3 times with 1 mL RIPA and bound proteins (biotinylated) were eluted with 2× Laemmli buffer (65 mM Tris, 10% glycerol, 2.3% SDS, 100 mM DTT, 0.01% bromophenol blue) for 10 min at 75 °C. Non-biotinylated and biotinylated fractions were then analyzed in Western blots.

### 4.6. Immunofluorescent Staining of Cultured Cells

Immunocytochemistry was performed as described [19]. Briefly, COS7 cells were fixed with 4% paraformaldehyde in PBS, washed three times with 1 mL of PBS, and then blocked for 1 h with 10% serum in TNT (0.1 M Tris/ HCl pH 7.5, 0.3 M NaCl, and 0.2% Triton X-100). The cells were then incubated for 2 h with the desired primary antibodies CNX (rabbit, 1:200, StressMarq, Victoria, BC, Canada, Catalog# SPC-108B), GFP (mouse, 1:100, Roche, Basel, Switzerland, Catalog# 11814460001), or RFP (rat, 1:1000, Chromotek, Planegg, Germany, Catalog# 5F8), Flag (mouse, 1:1000, Sigma-Aldrich, Catalog# F3165), HA (mouse, 1:1000, Covance/Biolegend, San Diego, CA, USA, Catalog# MMS-101P) or alfa2 NKATPase (rabbit, 1:200, Millipore, Burlington, MA, USA, Catalog# AB9094), diluted in TNT containing 1% serum (TNT-S), after which they were washed three times with TNT buffer and incubated for 2 h with the appropriate secondary antibody diluted in TNT-S. All secondary antibodies were from Thermo Fisher Scientific: anti-mouse Alexa Fluor 488 (1:500, Catalog# A-21202), anti-rabbit Alexa Fluor 555 (1:500, Catalog# A-31572), anti-rat Alexa Fluor 647 (1:500, Catalog# A-21247), and anti-rabbit Alexa Fluor 647 (1:500, Catalog# A-31573). After three washes with TNT, the cells were incubated for 10 min with DAPI (1:5000, Merck, Darmstadt, Germany, Catalog# 268298) diluted in TNT-S. After three new washes with TNT, the coverslips were mounted on microscope slides with Vectashield (Vector Laboratories, Burlingame, CA, USA), and the cells were visualized by confocal microscopy on the inverted microscope Nikon A1R+ (Nikon Instruments Inc., Tokyo, Japan) with high-sensitivity GaAsP detectors and six diode laser lines. At least 30 images for each condition were quantified using ImageJ 1.54k software (National Institutes of Health, Bethesda, MD, USA) [28]. The images were processed with a 2.0-pixel median filter (ImageJ 1.54k, National Institutes of Health), and the threshold applied was automatically determined by the JACoP plugin [69]. Pearson’s value of correlation was obtained with JACoP by comparing the two thresholded channels and measuring the correlation between them. The value can range from −1 to 1, the latter representing maximal correlation and colocalization (two identical images). To assess the organization of the actin cytoskeleton, the orientation of F-actin fibers was analyzed using the Directionality plugin in Fiji 2.9.0 (open-source software developed by the Fiji community) (ImageJ). Images of phalloidin-stained cells were first converted to grayscale, and individual cells were selected as regions of interest (ROIs). The analysis was performed using the Fourier components method, which computes the angular distribution of oriented structures in the image. The resulting Dispersion value reflects the degree of angular variability: lower Dispersion values indicate a higher degree of fiber alignment (i.e., more organized stress fibers), while higher values represent a more random or disorganized fiber orientation. This metric was used to compare the cytoskeletal architecture between cells expressing wild-type or mutant GlyT2.

### 4.7. Electrophoresis and Western Blotting

Protein samples were separated by SDS-PAGE using a 4% stacking gel and 6 or 7.5% resolving gels. The samples were transferred to nitrocellulose (Invitrogen, Carlsbad, CA, USA) at 1.2 mA/cm^2^ for 2 h. The membranes were blocked for 4 h with 4% non-fat dry milk (Central Lechera Asturiana, Asturias, Spain) in PBS at 25 °C and probed overnight at 4 °C with the desired primary antibody: anti-GlyT2 (home-made, rabbit, 1:500) [38]; anti-GlyT2 (home-made, rat, 1:200) [39]; mouse anti-calnexin (1:1000, BD Biosciences, San Jose, CA, USA, Cat# 610523, RRID:AB_39788); anti-ubiquitin (P4D1, 1:200, Santa Cruz Biotechnology, Dallas, TX, USA, Cat# sc-8017, RRID:AB_628423); anti-HA (monoclonal antiserum 12CA5, 1:500; Sigma-Aldrich, RRID:AB_514505); anti-GFP (green fluorescent protein, 1:1000; Invitrogen); or anti-RFP (red fluorescent protein, 1:2000, a generous gift of José María Requena (CBM)). After several washes with PBS-Tween 20 (Sigma-Aldrich, Catalog# 8.22184.0500) at 0.05%, the antibodies bound were detected with peroxidase-coupled anti-rat (1:8000; Sigma-Aldrich, Cat# A-5795), anti-rabbit IgG (1:8000; Bethyl, Montgomery, TX, USA, Cat# A120-401P) or peroxidase-coupled anti-mouse IgG (1:8000; Pierce, Appleton, WI, USA, Cat# 31452). Bands were visualized by enhanced chemiluminiscence (ECL, Bio-Rad, Hercules, CA, USA, Catalog# 160-5070). Linear-range film exposures were imaged using a GS900 calibrated imaging densitometer (Bio-Rad) and quantified using Image Lab Software 6.1 (Bio-Rad). Subsequently, the antibodies were stripped from the membrane (Thermo Fisher Scientific), which was reprobed with anti-α-tubulin (1:3000, Sigma-Aldrich, Cat# T6074, RRID:AB_477582), and antibody binding was detected with a peroxidase-coupled anti-mouse IgG.

### 4.8. Carbohydrate Modification

COS7 cells expressing the desired transporters were lysed in 1× lysis buffer (150 mM NaCl, 50 mM Tris-HCl pH 7.4, 5 mM EDTA, 1% Triton X-100, 0.1% SDS, 0.25% sodium deoxycholate, 0.4 mM PMSF, and 4 μM pepstatin) and digested with the chosen endoglycosidase, peptide: N-glycosidase F (New England Biolabs, Ipswich, MA, USA) or endoglycosidase H (Roche Applied Science, Penzberg, Germany), in a small volume of the appropriate buffer according to the manufacturer’s instructions. Cell extracts were resolved by SDS-PAGE and analyzed in Western blots [19].

### 4.9. Immunoprecipitation Assays

Transfected COS7 cells were washed twice with PBS and scraped off from the plates in 150 mM NaCl, 50 mM Tris-HCl pH 7.4, 0.4 mM PMSF, and 4 μM pepstatin, and the desired amount of protein determined by the Bradford method (Bradford Protein Assay Dye Reagent Concentrate, Bio-Rad, Catalog# 5000001) was solubilized for 30 min at 22 °C in lysis buffer with 0.2% Nonidet P-40. After 15 min centrifugation at 10,000× *g*, an aliquot of the lysate was retained to measure the total protein content, and the remainder was precleared by adding 20 μL of 50% protein G-Sepharose (PGS, Neo Biotech, Seoul, Republic of Korea, Catalog# NB-45-00037-5) in lysis buffer for 30 min at 4 °C with rotation. After centrifugation, the supernatants were incubated overnight at 4 °C with 2 μg of the desired primary antibody, whereas controls without the antibody were also included. Subsequently, 20 μL of protein G-Sepharose beads were added to the samples, and after incubating for 1 h at 4 °C, the beads were washed twice for 5 min with 500 μL of 1× lysis buffer. Bound proteins were then dissociated from the beads by heating at 75 °C for 15 min, resolved by SDS-PAGE, and analyzed in Western blots [19].

### 4.10. Isolation of Detergent-Resistant Membranes

Membrane domains resistant to the solubilization by Triton^TM^ X-100 (lipid rafts) were isolated as described [70]. Cell cultures were scrapped in 50 mM Tris-HCl pH 7.5 containing 150 mM NaCl and the protein concentration was determined by the Bradford method. In all subsequent steps, samples were kept at 4 °C. Equal amounts of protein from each sample were taken, and the cells were lysed in 50 mM Tris-HCl pH 7.5, 150 mM NaCl, 5 mM EDTA and 1% Triton X-100 for 40 min in rotation. Cells were centrifuged at 3000 rpm for 10 min and the precipitate was discarded. The supernatant was centrifuged in a Beckman ultracentrifuge (TL-100) for 1 h at 4 °C at 100,000× *g*. Finally, the supernatant (soluble fraction) and the precipitate (DRM fraction) were taken separately, included in Laemmli loading buffer, and analyzed through Western blots.

### 4.11. Ubiquitination Assay

COS7 cells treated with 10 µM MG-132 (Hözel Biotech, Köln, Germany, Cat#: HY-13259C) for 3–4 h at 37 °C were 2× washed with PBS at 4 °C and harvested using Ub buffer (50 mM Tris-HCl pH 7.5, 150 mM NaCl, 1 mM EDTA and 50 mM N-ethyl maleimide, NEM, with PI), and cell protein content determined [71]. Equal amounts of protein were centrifuged, and pellets were resuspended in 90 µL of Ub buffer. Then, 10 μL of 10% SDS were added, and samples were incubated for 10 min at 95 °C to disrupt protein interactions. Afterwards, samples were diluted by adding 34 μL of Ub buffer containing 4% Triton X-100 and 1 mL of Ub buffer containing 1% Triton X-100. After 30 min on rotary shaking at 4 °C, lysates were precleared with 50% Protein G-sepharose (PGS, Neo Biotech, Seoul, Republic of Korea, Cat#: NB-45-00037-5) in Ub buffer for 30 min at 4 °C and then overnight incubated with the anti-GlyT2 antibody. Then, PGS was added for 90 min at RT followed by 3 washes with ice-cold Ub buffer and elution in 2× Laemmli buffer at 75 °C for 15 min. Samples were subjected to 6% SDS-PAGE and WB with ubiquitin-specific antibodies and GlyT2 antibodies.

### 4.12. Proteomics

#### 4.12.1. Preparation of Protein Extracts and Protein Digestion

Proteins were extracted from the cell pellets by boiling in the presence of extraction buffer (50 mM Tris-HCl pH 6.8, 2% sodium dodecyl sulfate, 10 mM dithiothreitol) for 5 min. After centrifugation, the protein content was measured using the RC DC protein assay (Bio-Rad), and thereafter, the proteins were loaded onto FASP filters (Expedeon, San Diego, CA, USA) for protein digestion, which was carried out overnight at 37 °C with sequencing grade trypsin (Promega, Madison, WI, USA) at a 1:40 (*w*/*w*) trypsin/protein ratio following the manufacturers’s instructions. The resulting tryptic peptides were recovered by centrifugation, after which trifluoroacetic acid (TFA) was added to a final concentration of 1% and the peptides were desalted on C18 Oasis HLB extraction cartridges (Waters Corporation, Milford, MA, USA). The resulting peptide solutions were vacuum-dried.

#### 4.12.2. Isobaric Labeling of Tryptic Peptides

The peptide samples were taken up in 1 M triethylamonium bicarbonate and their concentration was determined using a Direct Detect IR spectrometer (Millipore, Billerica, MA, USA). Equal amounts of the resulting peptides were isobarically labeled with tandem mass tag (TMT) 10-plex reagents (Thermo Fisher Scientific) following the manufacturer’s instructions. The resulting labeled peptide samples were pooled, and the resulting mix was vacuum-dried.

#### 4.12.3. Liquid Chromatography–Tandem Mass Spectrometry Analysis

The labeled peptide samples were taken up in 0.1% formic acid (FA) and subjected to liquid chromatography–tandem mass spectrometry (LC-MS/MS) analysis using an Easy nLC 1000 nano-HPLC (Thermo Fisher Scientific) coupled to an Orbitrap Fusion tribrid mass spectrometer (Thermo Fisher Scientific). The peptides were loaded onto a PepMap 100 C18 LC pre-column (75 µm internal diameter, 2 cm length, Thermo Fisher Scientific) and resolved on a NanoViper PepMap 100 C18 LC analytical column (75 µm diameter, 50 cm length, Thermo Fisher Scientific) using a linear gradient of buffer B (8–31% in 5 h; B, ACN 90%/FA 0.1%) at a 200 nL/min flow rate. Mass analysis was carried out following a data-dependent acquisition method: the full scan of precursor ions was performed in the 390–1500 Th range using 5 × 10^5^ automatic gain control (AGC) and 50 ms maximum injection time at 120,000 resolution. Precursor ions were then isolated based on their intensity in a Top 20 mode to induce their fragmentation by higher-energy collisional dissociation (HCD) using a normalized collision energy of 33%. The fragments thus generated were detected in the Orbitrap analyzer with 30,000 resolution. The precursor isolation window in the quadrupole was set to 1.5 Th and the dynamic exclusion was set to 40 s.

#### 4.12.4. Peptide and Protein Identification

For peptide identification, the fragmentation spectra were analyzed using the SEQUEST HT search engine (Thermo Fisher Scientific) [72] implemented in the Proteome Discoverer 2.5 program (Thermo Fisher Scientific) [73]. The assignment of peptide sequences was carried out by matching the experimental mass data against the Chlorocebus sabaeus Uniprot protein database (as of June 2023) supplemented with the rat GlyT2 sequence. The following search parameters were used: tryptic digestion with two maximum missed cleavage sites; 800 ppm and 0.02 Da precursor and fragment mass tolerance, respectively; Cys carbamidomethylation (+57.02146) and N-terminus and Lys TMT labeling (229.16293 Da) as fixed modifications; and variable Met oxidation (+15.99492). The corresponding inverted protein sequences were incorporated into the database for subsequent estimation of the false positive rate (FDR) for peptide identification, which was calculated using the probability ratio method with 15 ppm precursor ion mass tolerance postfiltering [74]. A 1% FDR threshold was considered for the identification of peptides, which were assigned to the most probable protein proposed by Proteome Discoverer. The mass spectrometry proteomics data were deposited to the ProteomeXchange Consortium via the PRIDE [75] partner repository with the dataset identifier PXD061845.

#### 4.12.5. Statistical Assessment of Protein Abundance Changes

The quantification of peptide and protein abundance were performed using iSanXoT 2.0.0 (CNIC, Madrid, Spain) [76], a publicly available implementation of the WSPP statistical model and the Generic Integration Algorithm [77]. Protein log2-ratio values were expressed in units of standard deviation according to their estimated variance (Zq values). The Limma package 3.22 (Walter and Eliza Hall Institute of Medical Research, Parkville, Australia) [78] was used to ascertain statistically significant protein abundance changes between cells expressing the wild-type and mutant proteins. To interpret protein abundance data, interaction network and enrichment analysis was performed using STRING [42].

### 4.13. Statistical Analysis and Data Representation

A statistical analysis of the data and graph representation was performed using GraphPad Prism 7 (Dotmatics, Boston, MA, USA, RRID:SCR_002798). Student’s *t* test was applied to compare two experimental groups. Multiple comparisons of different conditions were performed using a two-tailed one-way analysis of variance (ANOVA) with Sidak’s or Dunnett’s multiple comparison test. *p*-values lower than 0.05 were considered statistically significant and were represented as follows: *p* < 0.05 (*), *p* < 0.01 (**), *p* < 0.001 (***), and *p* < 0.0001 (****). The absence of asterisks indicates that no significant differences were detected. No test for outliers was conducted. Mean values along with the standard error of the mean (SEM) of at least 3 experiments were represented in the graphs.

## Data Availability

The datasets generated and/or analyzed during the current study are not publicly available due to individual privacy, but some data are available from the corresponding author on reasonable request. Proteomic data are available via ProteomeXchange with the identifier PXD061845.

## Supplementary Materials

The supporting information can be downloaded at https://www.mdpi.com/article/10.3390/ijms26146753/s1.

## Abbreviations

The following abbreviations are used in this manuscript:

CNX	calnexin
COPII	coatomer protein II
DRM	detergent-resistant membrane
ERAD	ER-associated degradation
GlyT	glycine transporter
GlyR	glycine receptor
GLR	glycine receptor subunit
LeuTAa	leucine transporter from *A. aeolicus*
NAG	N-arachidonoyl glycine
PBA	4-phenylbutyric acid
SLC	solute carrier
TM	transmembrane domain
