# Supplementary material for: A New GlyT2 Variant Associated with Hyperekplexia
Source: Int J Mol Sci. 2025 Jul 14;26(14):6753. doi: 10.3390/ijms26146753 (PMC12295724; doi:10.3390/ijms26146753)
Supplement: Supplementary file 1 [file ijms-26-06753-s001.zip › ijms-3676436-supplementary/Supplementary Figures.pdf]

Figure S1

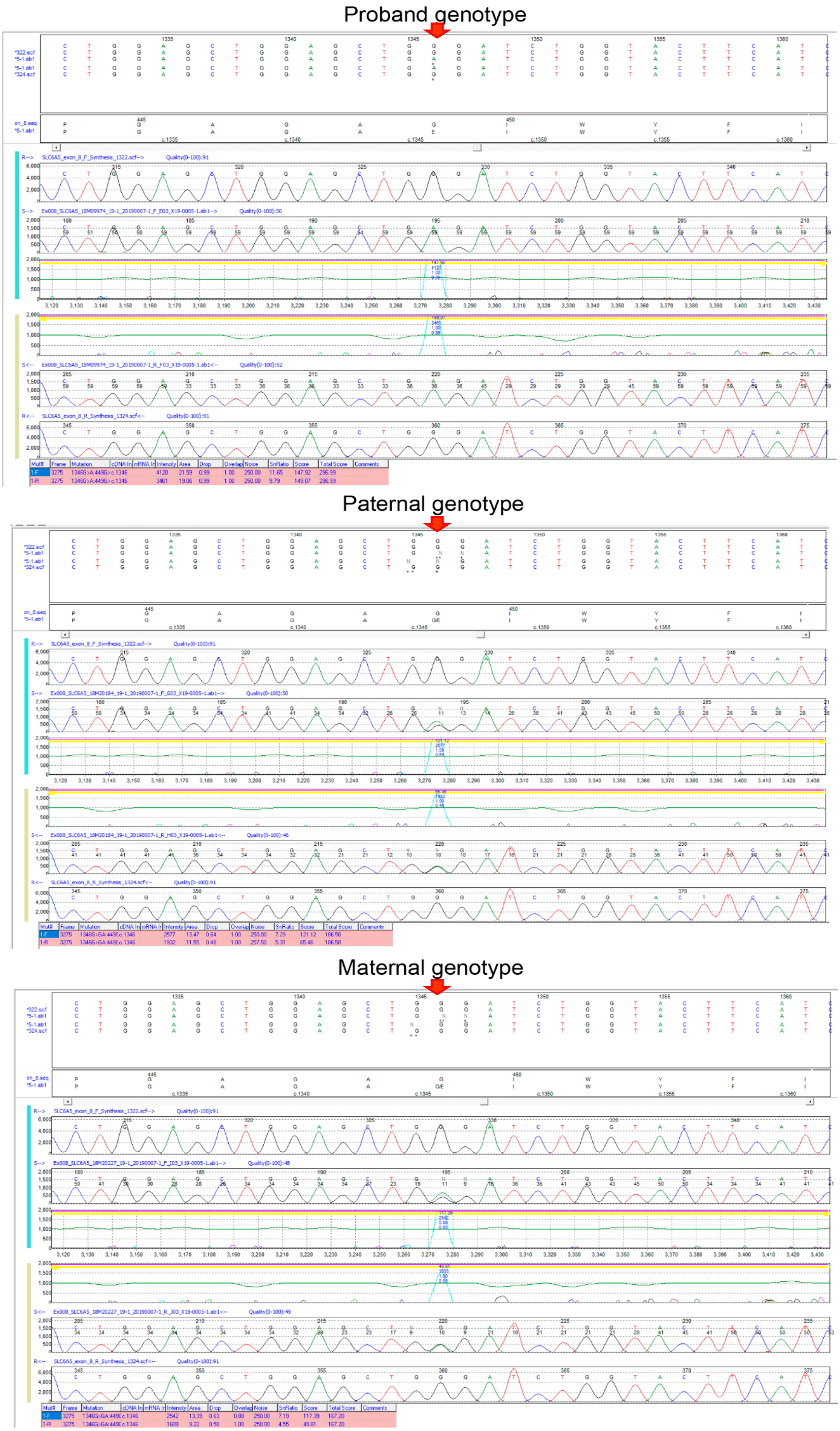

Figure S1. Screenshots of the proband and parental Sanger sequencing data. A genomic DNA sample was scanned on all 16 coding exons and extended flanking intronic regions of SLC6A5 (11p15.1), encoding human GlyT2. Sanger sequences of exon 8 of the SLC6A5 gene in the proband, his father and mother are shown. The proband was found to have a single homozygous substitution - c.1346G>A, which appeared in heterozygosity in the parents.

Figure S2

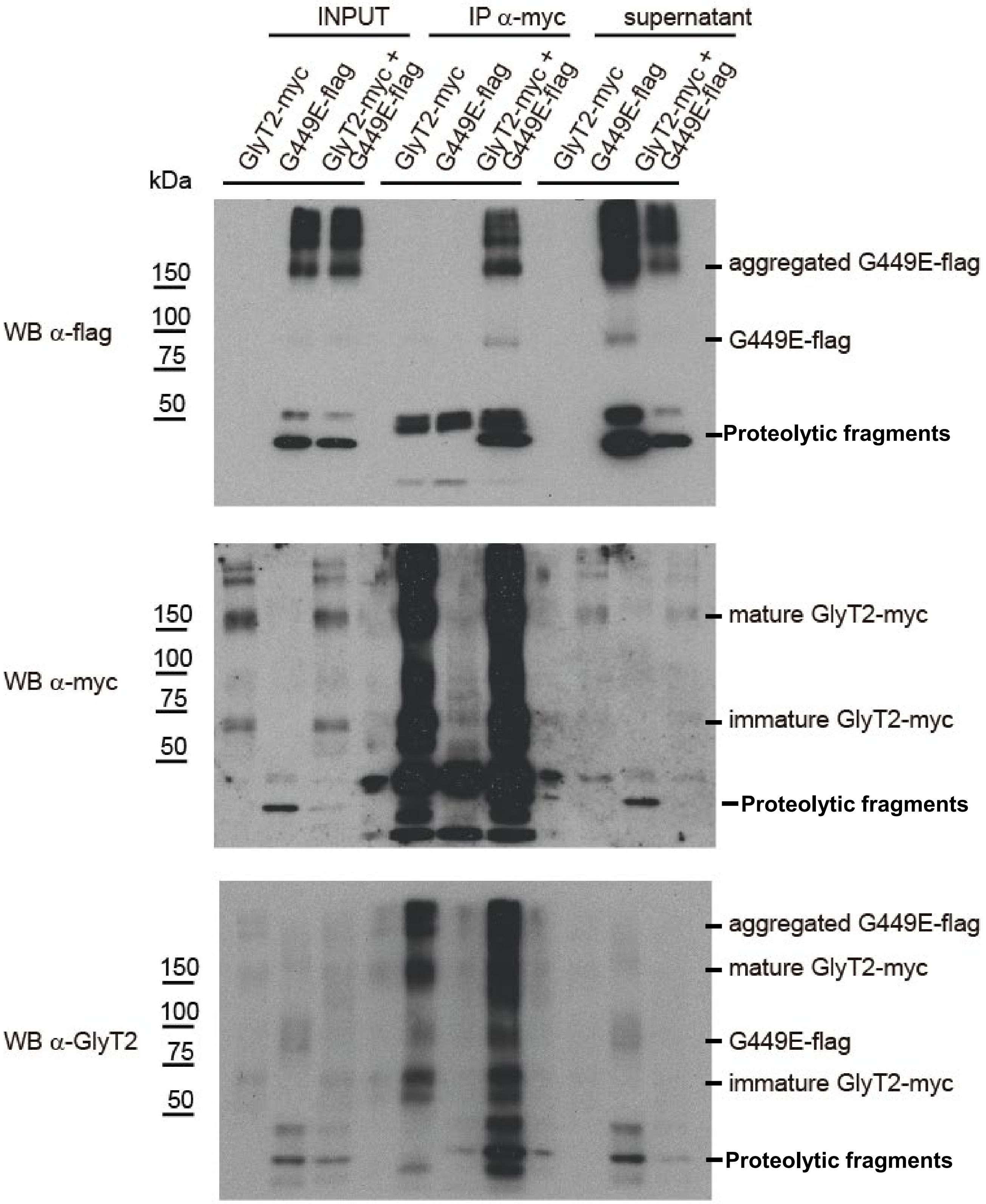

Figure S2. Co-precipitation of wild-type and mutant transporters. Lysates of COS7 cells expressing the indicated differentially tagged transporters were immunoprecipitated with anti-myc antibody, and the immunocomplexes were analyzed in Western blots to detect myc, flag, and GlyT2.

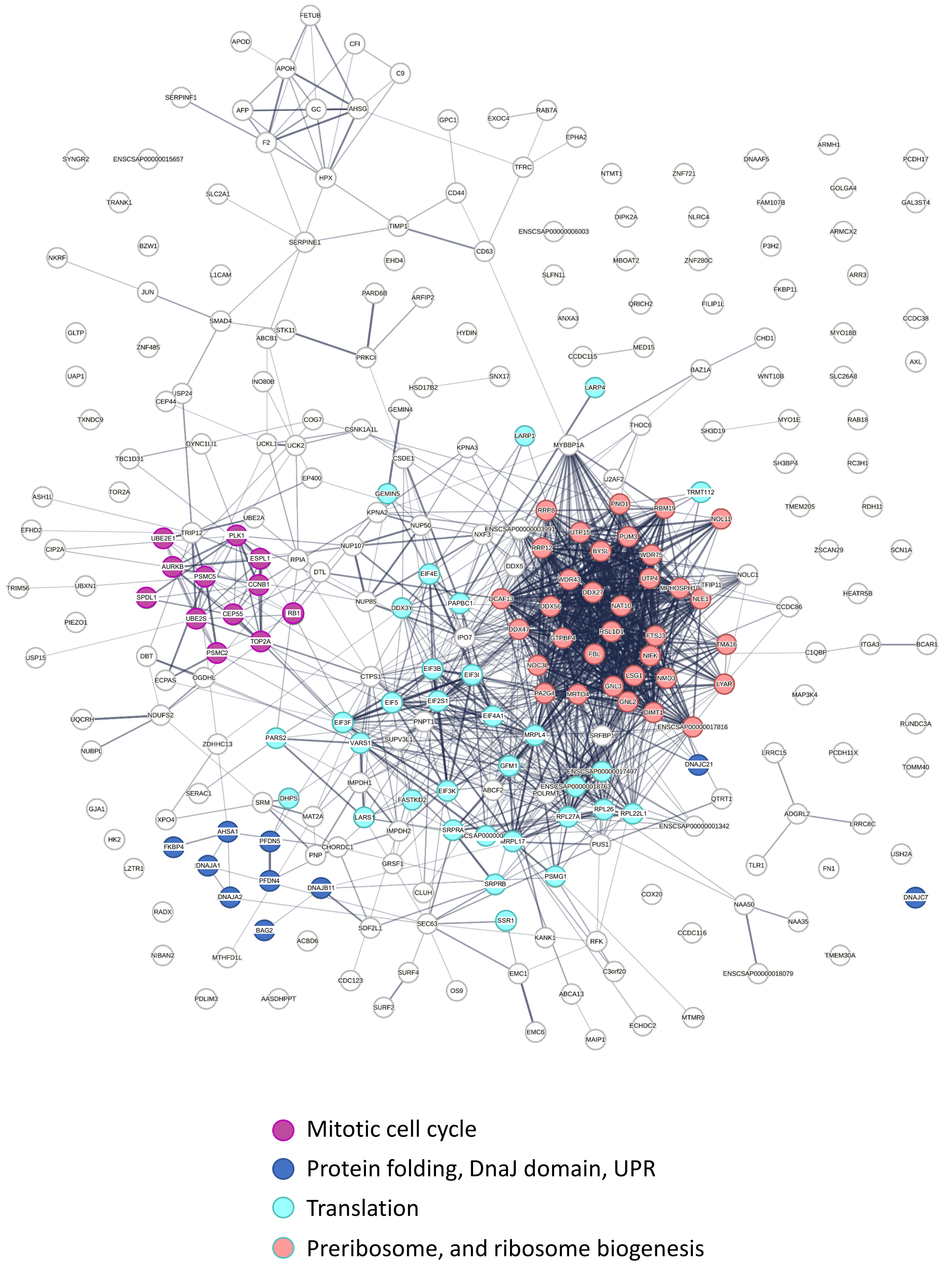

Figure S3: STRING interactome of the main significantly enriched categories seen in the cells expressing the G449E mutant as compared to the cells expressing the wild-type.

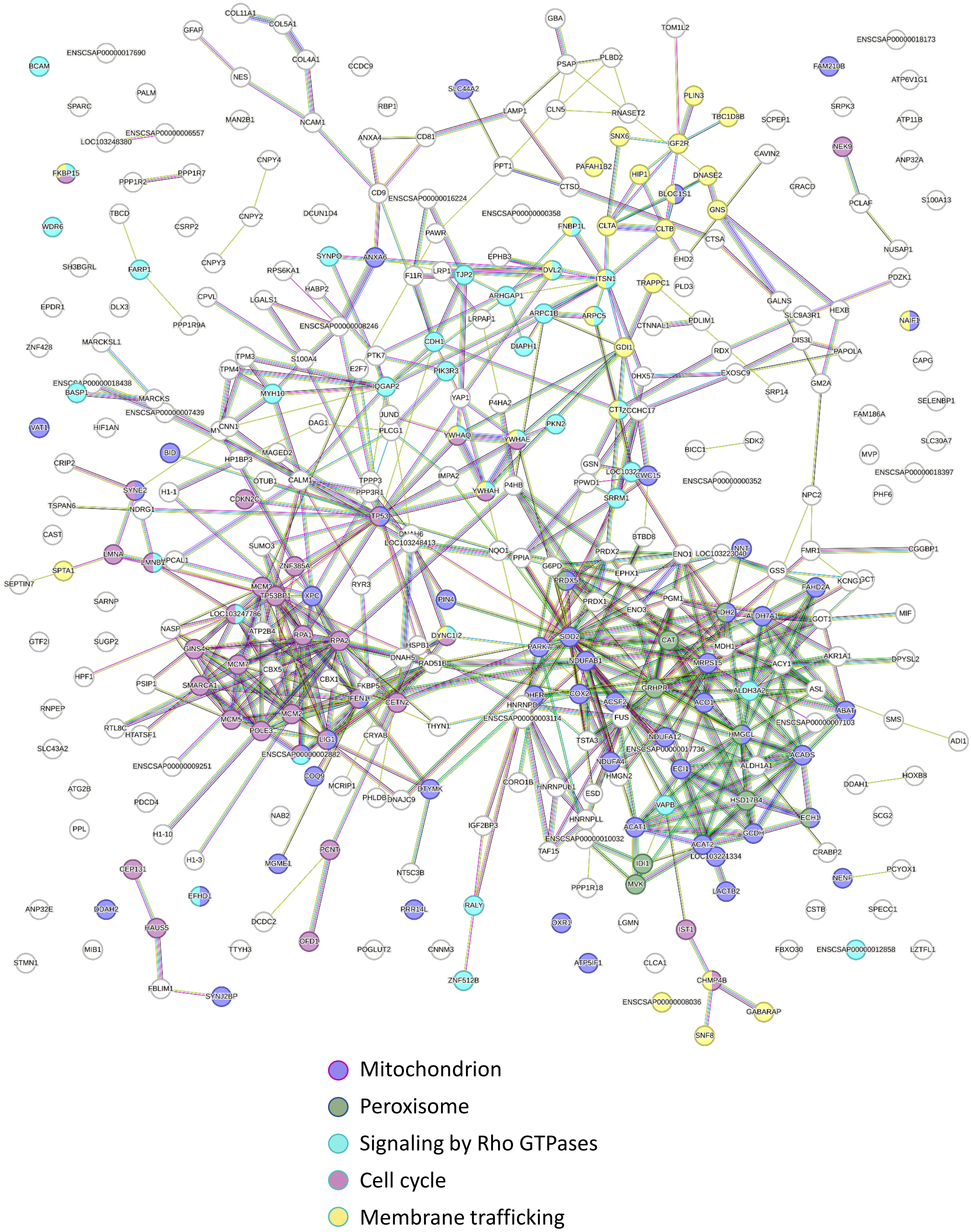

Figure S4: STRING interactome of the main significantly down regulated categories seen in the cells expressing the G449E mutant as compared to the cells expressing the wild-type.

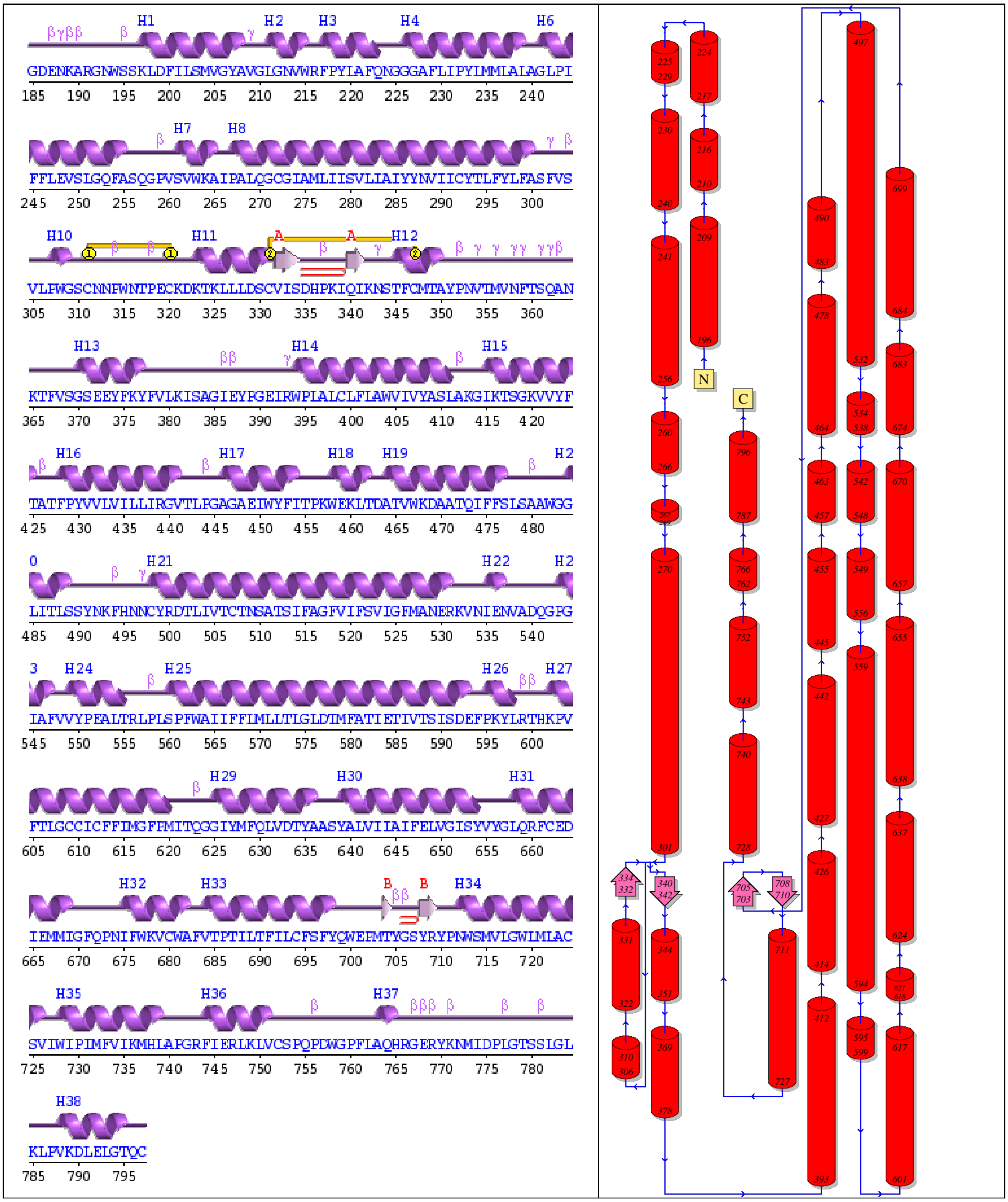

Figure S5. **Left.** Schematic diagram showing the sequence and corresponding secondary structure elements of the 3D molecular model of the human GlyT2 E449E variant studied (residues 185-797). Helical regions (labeled H1, H2, etc.) and b-strands are represented by helices and arrows, respectively. Beta and gamma turns are indicated with Greek letters. Yellow circles and bars represent disulfide bridges. **Right.** Topology diagram of the wild-type human GlyT2 3D molecular model.
